# Higher Education Students’ Reflective Journal Writing and Lifelong Learning Skills: Insights From an Exploratory Sequential Study

**DOI:** 10.3389/fpsyg.2021.707168

**Published:** 2022-01-07

**Authors:** Dorit Alt, Nirit Raichel, Lior Naamati-Schneider

**Affiliations:** ^1^Kinneret College on the Sea of Galilee, Kinneret, Israel; ^2^Hadassah Academic College, Jerusalem, Israel

**Keywords:** reflective journaling, lifelong learning, metacognitive reflective scaffolds, formative assessment, higher education

## Abstract

Reflective journal (RJ) writing has been recognized as an effective pedagogical tool for nurturing students’ lifelong learning skills. With the paucity of empirical work on the dimensionality of reflective writing, this research sought to qualitatively analyze students’ RJ writing and design a generic reflection scheme for identifying dimensions of reflective thinking. Drawing on the theoretical scheme, another aim was to design and validate a questionnaire to measure students’ perceptions of their reflective writing experiences. The last aim was to quantitatively measure the link between perceived reflective writing and students’ tendency to use RJs in their future careers and personal lives. This exploratory sequential research included the following steps: First, experts’ review and analysis of 1312 RJ entries were attained. This step led to the design of a theoretical scheme of reflective writing and a 31-item questionnaire, used to gather data from 171 students (second-year pre-service teachers and third-year health managers). Partial Least Squares analysis corroborated the structure suggested by the theoretical scheme: two timelines–reflections regarding the current course assignments and those related to the student’s future development. Students’ tendency to use reflective skills in their future professional lives was highly connected to their long-term reflections, including learning experiences linked to academic, professional, personal, and multicultural development. The current study’s suggested validated generic scheme can be adapted and integrated into different curricula, thereby possibly increasing the potential of infusing RJ instructional strategies into higher education curricula, improving the quality of reflection in student journals, and promoting lifelong learning skills.

## Introduction

The ability to reflect on one’s learning is a fundamental skill necessary for lifelong learning (Ryan, 2015). Therefore, encouraging students to engage in reflective practices has been acknowledged as an essential goal in higher education for effectively preparing students for their subsequent professional experiences (Adie and Tangen, 2015). While the potential of reflective practices to engender lasting and effective changes in students’ lives is widely recognized (Waggoner-Denton, 2018), a somewhat opaque landscape emerges in terms of identifying the dimensionality of reflection and the potential connection between using reflective writing and subsequent adoption of reflective practice into students’ personal or professional lives (Griggs et al., 2018). Indeed, while some previous investigation has explored the issues of measuring reflection and assessing student reflective journal (RJ) writing (e.g., Kember et al., 2008), a widely accepted method for identifying and assessing reflection does not exist (Waggoner-Denton, 2018).

To address these issues, the present research sought to analyze pre-service teachers’ and health managers’ RJ writing and design a generic reflection scheme for identifying dimensions of reflective thinking. Drawing on the theoretical scheme, another aim was to design and validate a questionnaire to measure students’ perceptions of their reflective writing experiences. The last aim was to quantitatively measure the links between perceived reflective writing and students’ tendency to use RJs in their future professional and personal lives.

This exploratory sequential mixed-methods study aims to make two contributions to higher education literature on reflective practices. The first is to design and validate a comprehensive and generic practical scheme for prompting questions designed to promote future professionals’ higher-order thinking skills by using written journals. The second is to provide insights into how pre-service teachers and health managers reflect upon their learning and how they perceive this experience. This study could also shed light on the potential connections between perceived reflective learning processes and the tendency to use skills acquired during these processes in future professional and personal settings.

## Literature Review

### Reflectivity: A License for Lifelong Learning

Lifelong learning is an approach whereby the individual engages in a continuous process of adjusting his/her capabilities in relation to the changing expectation of the work and learning environments (Archer, 2007). From this stems the broad definition of lifelong learning as learning activities experienced throughout life aimed at improving “knowledge, skills and competence, within a personal, civic, social and/or employment-related perspective” (Commission of the European Communities [CEC], 2001, p. 9). This definition relates to all areas of life that could be relevant to the individual’s and society’s coping with 21st-century challenges.

Arguably, in the current fluid and changing conceptual age (McWilliam, 2017), individuals must actively manage their capabilities in a meaningful manner. Individuals who are able to reorient themselves in new ways are more likely to have a sense of agency (Ryan, 2015). Effective choices require a reflective approach to learning; hence, lifelong learning generally necessitates self-regulation and self-assessment. Reflection, which is regarded as a formative self-assessment, is deemed essential in contemporary higher education settings. Self-assessment is our utilization of the information and feedback we receive from multiple sources. Schön (1983) maintained that through reflection, the practitioner can make new sense of uncertain situations.

### Reflective Journals

One active learning method designed to promote higher-order thinking skills is the RJ, also referred to as learning diaries/journals, or learning/response logs. RJ is perceived as a vehicle for reflection (Moon, 2006). RJs were defined as “written documents that students create as they think about various concepts, events, or interactions over a period of time for the purposes of gaining insights into self-awareness and learning” (Thorpe, 2004, p. 328). Journal writing is related to metacognition as it, “slows the pace of learning, increases the sense of ownership of learning, and… has been described as a bridge across which learners can move from the specific to the general, while developing a habit of reflection” (Cowan, 2014. p. 54).

This tool aims to express the self-observation of the learning process and evidence of reflection (Wallin and Adawi, 2018). An essential benefit of this assessment tool is the creation of an environment where students feel encouraged and safe to express their concerns and explore their thoughts, to reflect on their beliefs, values, experiences, and assumptions that influence their learning, as well as their development and progress over time (Minott, 2008). RJs can also affect the behavior, as indicated by Fabriz et al. (2014), during the reflection stage, the learners evaluate their work and judge if the goal has been attained. Following this judgment, they react and regulate their behavior with regard to further learning processes. Thus, the outcome of a prior learning process informs the subsequent learning process.

Indeed, research into the use of RJs to encourage higher-order thinking skills is a growing field in higher education research and practice (Bell et al., 2011). Therefore, engaging students in reflective practices has been recognized as a central goal for learning and transformation and for preparing students effectively for their future professional lives (McCarthy, 2011; Ryan, 2015); for improving students’ lifelong learning and professional practice in higher education (Ryan, 2011); for effectively functioning in a diverse and complex practice environment (McGuire et al., 2009) and; for gaining pedagogical insights from learning activities (Hume, 2009).

While previous work on critical reflection is fragmented and lacks a clear and widely accepted definition, some studies have explored the issues of measuring reflection and evaluating student RJs. For example, drawing on previous work (Boud et al., 1985; Mezirow, 1991), Wong et al. (1995) designed a protocol that required identifying instances of the use of elements such as attending to feelings, association, and integration. However, it was argued by Kember et al. (2000, 2008) that employing this scheme is challenging for those unfamiliar with the literature. Therefore, they developed criteria to evaluate the existing coding schemes employed to assess students’ reflective practice in a nursing education context. Their first attempt to develop a protocol, in which the categories were too fine-grained, led to the developing of a simpler scheme with fewer categories. A questionnaire they developed to measure levels of reflective thinking was designed and then validated with four scales: habitual action/non-reflection, understanding, reflection, and critical reflection. To achieve consistency between the quantitative and qualitative methods of determining levels of reflection, a four-category scheme was developed and recommended to be performed at the whole-journal level to ascertain the highest level of reflection of each individual student (Kember et al., 2008).

Several studies have employed this coding scheme (Thorpe, 2004; Samkin and Francis, 2008; Bell et al., 2011), using a small sample size of students in only a few disciplines. For example, Bell et al. (2011) evaluated the validity of this coding scheme in a business education context, by coding students’ RJ entries based on the proposed scheme. It should be noted that the assessment process required three independent coders. Each coder was familiarized with the theoretical framework of Kember et al.’s (2000; 2008) coding scheme. The coders then independently coded one page of the entry. The codes allocated by the coders were compared. Yet, the authors asserted that it may not be practical to do such detailed coding with a larger cohort of students. Moreover, they argued that it is not appropriate to use the scheme as a mechanism for assessment, and they questioned the grading process by contending, for example, that the highest level of reflection does not necessarily represent the broad spectrum of reflection that may be encompassed in the student’s writing.

While in the studies mentioned above unstructured reflective writing was assessed (i.e., in which students were simply prompted to “reflect” on their performance without detailed guidance on what to include in their entries) other researchers such as Waggoner-Denton (2018), who incorporated a reflective learning journal into an introductory statistics course, pointed out a major challenge in this learning method, for researchers and students alike. Some students, they argued, may find the writing process to be particularly burdensome. To circumvent this problem, it was suggested to provide students with more specific prompts for their journal entries (Learning to Learn Project, 2002), for example, “what strategy have I used in learning this topic?” or “what is learning?” These structured journals may assist students in recognizing difficulties and prevent more personal reflection from occurring (Waggoner-Denton, 2018). Self-monitoring prompts help students think about their learning approaches and processes, thereby making them visible (English and Kitsantas, 2013; Moussa-Inaty, 2015; Wallin and Adawi, 2018).

Other researchers also underscored the effectiveness of using prompts in RJs. For example, Molee et al. (2011) assessed students’ depth of learning and critical thinking through reflection in service-learning courses at a public university. Prompting questions were used to help students address their academic enhancement, personal growth, or civic engagement as they examine each specific learning objective in their reflection process. The participants followed specific prompts to guide their reflections. For example, they were asked to provide objective descriptions of their experiences by answering questions such as Who? What? Where? When? Analytic evaluations were prompted by asking the students to make a reasoned judgment. The researchers also used questions to help students revise their reflections (e.g., “what goals shall I set in accordance with what I have learned, to improve myself, the quality of my learning, or the quality of my future experiences or service?” p. 243). Based on their study, prompting questions were found useful in documenting student learning. Yet, students had difficulty evaluating their learning and thinking critically. The authors ascribed these limitations to the novelty of the tool and to participants’ insufficient experience with reflection processes.

Similarly, Krauskopf et al. (2018) investigated the meta-cognitive awareness of teachers’ Technological Pedagogical Content Knowledge (TPACK). The authors supported the notion that prompting teachers to reflect on the personal perceptions of their professional knowledge might improve their performance in lesson designing. Prompting questions stimulated reflective discussion, engaged the participants in meta-cognitive development, and enabled them to create subjective interpretations of their knowledge, thus supporting their individualized growth. The findings suggested that the process aided teachers in reflecting on their professional knowledge and in determining their own professional development. In a similar vein, Verpoorten et al. (2012) assessed the effects of prompting questions in an online course. The structured opportunities for reflection were perceived as useful to reflection and learning. The authors suggested nesting prompts in the study material during learning activities to induce ongoing mental tingling for self-evaluation.

### The Dimensionality of Reflective Writing

Drawing on metacognition theory, the Learning to Learn Project (2002), and recently the Assessment Tools for Higher Education Learning Environments (Assessment Tools for Higher Education Learning Environments [ASSET], 2020) ERASMUS+ project teams created a bank of prompt questions systematically organized around three dimensions of essential metacognitive abilities for independent, reflective learners. These dimensions of metacognitive reflection are awareness, evaluation, and regulation (Brown, 1987; Jacobs and Paris, 1987; Magiera and Zawojewski, 2011). Awareness, or metacognitive knowledge, is a state in which the individual is aware of what s/he knows (tasks, specific knowledge). Evaluation is a process in which a person is thinking about the effectiveness and limitations of his or her mind, and the effectiveness of his/her chosen strategy. Metacognitive regulation is a state in which a person thinks about his/her strategic planning and goal setting and involves the actions s/he takes in order to learn (Sandi-Urena et al., 2011; Purnomo and Bekti, 2017). While there is considerable literature on metacognitive abilities for reflective learners, scholars (e.g., Archer, 2010) argued that reflective tasks often exclude affective dimensions of learning and are mainly focused on the cognitive level rather than the emotional one. As students learn in different ways in diverse learning styles, it is important to enable them to reflect on their own learning styles, skills, situation, and motivations.

In addition to the cognitive and affective levels, for learning to produce ongoing benefits for both the learner and the work or learning environment, it must involve the learner’s active engagement. Reflective learning processes should prompt a deliberative action. Critical reflection is only achieved if action ensues. Metacognitive thinking skills alone will fail to address the social contexts and structures that influence learning. Thus, these processes should include a reflective interplay between individuals and social structures to understand and change courses of action chosen by individuals (Ryan, 2015).

In contrast to the numerous studies on the topic of metacognitive reflection dimensions in science education (Magiera and Zawojewski, 2011; Zohar and Barzilai, 2013; Stanton et al., 2015), there is a dearth of empirical work related to these components in the context of reflective writing in teacher education. The importance of incorporating reflection into teacher education is highlighted for example by Bailes et al. (2010), who asserted that complex situations often occur in schools, and the need to engage in lifelong learning underlies the rationale for integrating reflection into teacher education. The review of literature since 1995 of reflective practice in teacher education (Standal and Moe, 2013) which pointed to pre-service teachers’ inability to reflect critically, substantiates the call to use reflection in teacher training programs.

Metacognitive reflection dimensions are also considered core competencies of healthcare professionals (Lajoie et al., 2019). Medical residents are expected to identify their own knowledge gaps and to seek help from supervisors when they need it (Bransen et al., 2020). To this end, they must continuously define their own learning needs, set personal goals, and engage in the most precise and appropriate learning activities for them. It means, in essence, that they have to self-regulate their learning and become metacognitively active participants in their own learning processes (Lucieer et al., 2016). Therefore, self-regulated learning (SRL) is considered a core competence essential to the safeguarding of patient care (Sandars and Cleary, 2011; Ericsson, 2015; Alt and Naamati-Schneider, 2021).

Despite the importance of these reflection components, students often struggle to regulate their learning in clinical learning environments due to the unpredictable and dynamic nature of clinical workplace environments (Lucieer et al., 2016; Bransen et al., 2020). When they commence the clinical part of training, students tend to experience difficulties while interacting with patients and medical staff. In addition, they are required to transfer what they have learned to the workplace setting, to this end, they need to learn new material by using self-directed approaches to learning. In the early years of medical practice, medical staff bears many responsibilities related to the delivery of patient care. These are accompanied by an increased number of tasks initiated and performed independently. Hence aside from providing safe and efficient patient care, they are required to exhibit self-directed learning skills (Teunissen and Westerman, 2011). Therefore, healthcare curricula are increasingly called upon to support metacognitive reflection as a central learning outcome by employing reflective writing to enable students to give meaning to their learning experiences (Rajhans et al., 2020). The current study addresses one of such curricula–health systems management, aimed to train students at a high scientific and applied level in the fields of management based on knowledge and understanding related to public health, health systems, health policy and communication in therapist-patient relationships in clinical environments and clinical organizations. This training is necessary to produce graduates with integrated management and care abilities in this unique field. In recent years, the field of health systems management and health leadership development has evolved with the understanding that health systems and health organizations should be treated with a broader vision that includes a combination of clinical and managerial skills (Kuhlmann and von Knorring, 2014; Gilbert et al., 2019).

### Reflective Writing and Transfer of Learning

A central goal of education is to provide learning experiences that are useful beyond the specific conditions of initial learning (Marton, 2006; Lobato, 2012). These experiences should be accompanied by reflective thinking that might instigate new ideas and actions for improvement. This notion was forward by Kolb (1984) who offered a model of experiential learning and suggested that learners must first reflect before they can move onto active experimentation. The model includes four cyclical stages of experiential learning: concrete experience, reflective observation, abstract conceptualization, and planning active experimentation. According to Kolb (1984), “Learners […] must be able to reflect on and observe their experiences from many perspectives (RO). They must be able to create concepts that integrate their observations into logically sound theories (AC), and they must be able to use these theories to make decisions and solve problems (AE).” (p. 30).

Thus, the reflective practice encourages the learner to continue to learn from experience and bridge the gap between theory and practice and become a lifelong learner (Kolb, 1984). Based on this theory, Brown et al. (2011) argued that reflection should be considered a pre-requisite for transfer of knowledge and skills across classroom and work contexts and that incorporating a reflection tool into the training design could facilitate transfer. Similarly, other researchers (Asfeldt et al., 2018; Slade et al., 2019; Morris, 2020) underscored the importance of critical reflection in the process of learning and discussed its vital role as a mediator of meaning-making.

Griggs et al. (2018) examined the potential connection between reflective learning and the subsequent adoption of reflective practice in work. Their study examined RJs of 75 participants in a leadership development program, to assess their utility for facilitating transfer. Among other results, their findings suggested that organizational training can benefit from using reflective tools, such as RJs, to enhance transfer. The participants have been encouraged and taught to “see things differently” (p. 9) and critically evaluate other perspectives and recognize the complexity of reality. The combination of expert knowledge with instruction on how to use reflective tools contributed to the emergence of mature professionals relative to the perspectives they held prior to their professional program. The researchers argued, however, that the evidence to support and inform reflective practice in curriculum interventions remains largely theoretical. They stressed the lack of empirical data to indicate that the development of reflection in an academic context has long-term and definitively benefits most learners. Thus, in contrast to the plethora of literature on the teaching and learning of reflection (Hume, 2009; McGuire et al., 2009; Bailes et al., 2010; Brown et al., 2011; Dyment and O’Connell, 2011; McCarthy, 2011; Cowan, 2014; Adie and Tangen, 2015; Ryan, 2015), the dearth of evidence concerning transfer is inescapable.

Thus, arguably, when students are guided to reflect deeply on their learning, they are further encouraged to contextualize their learning in relation to their current academic and future professional lives (Dyment and O’Connell, 2011; Adie and Tangen, 2015). Based on this notion, another set of dimensions for questions to prompt students to engage in reflection processes was set by the Learning to Learn Project (2002) team. The first, “Explore a learning experience,” deals with the specific and the immediate. This helps improve students’ current performance. The second concerns lifelong learning skills or long-term issues, which help students recognize the relevance of their learning to their academic, professional, or personal development.

### Research Aims, Questions, and Hypotheses

Accompanying the lack of consensus regarding the optimal means of assessment of reflective practices is a paucity of empirical work on the dimensionality of reflective writing, in general, and in higher education, in particular. Therefore, this research sought to analyze students’ RJ writing, design a reflection scheme, and design and validate a questionnaire based on the scheme, aimed at measuring higher education students’ perceptions of reflective writing experiences. Moreover, unlike much of the existing research on RJ writing, this study sought to quantitatively measure the connection between perceived reflective writing and students’ tendency to transfer their acquired RJ writing skills to their future professional and personal lives, according to their own reporting. To this end, an exploratory sequential research design was employed in which the researcher begins with qualitative data and then collects quantitative information. This design is often used to identify themes, design an instrument, and subsequently test it (Creswell, 2012).

In line with previous research, asserting that activities that foster deep learning such as reflective writing may lead to greater transfer (e.g., Brown et al., 2011; Griggs et al., 2018), two hypotheses were evaluated. It was expected that students’ perceived reflective writing skills they have gained during the learning process will be positively connected to their perceived tendency to transfer RJ writing practices to their work environment–professional lives (*H1*), and to their personal lives (*H2*). An effort will be made to detect different trajectories within each sample group, as presented below.

## Materials and Methods

### Participants

Data were gathered from 141 students, of whom 75 undergraduate second-year Education students (pre-service teachers) from one major college located in northern Israel, and 66 undergraduate third-year students of Management of Health Service Organizations program from a central academic college in Israel. Students in this track are exposed to management studies and specific studies in the fields of environment and clinical care and are therefore required to develop relevant and specific abilities required in these fields, as part of adapting health systems to the needs of the changing professional requirements. Their extensive training enables the development of management and organizational skills alongside the development of personal vision as therapists in clinical environments. Most of the students in this track are therefore students who come from clinical therapeutic fields and are part of the health system and have a background and experience in clinical organizations, and they seek to advance and expand their academic knowledge beyond the therapeutic field to the administrative field. Such an integrated degree enables advancement in the field while having a broad multidisciplinary vision, taking into account systemic considerations.

Eighty-one percent of the students were females. The distribution regarding ethnicity was: 23.6% Jewish students, 75% Muslim students, and 1.4% Christian students (Arabic native speakers), with a mean age of 25.58 (*SD* = 6.89) years. The questionnaire was submitted to them by the end of the course. Prior to obtaining participants’ written consent, it was specified that the questionnaires were anonymous, and the participants were assured that no specific identifying information about the courses would be processed. The research was approved by the college’s Ethics Committee.

### Procedure

The procedure included the design of a new measurement to assess students’ perceptions of reflective writing experiences. RJ was used in two courses (see descriptions below). Experts’ review and analysis of the RJ entries were attained. This step led to the design of a theoretical scheme of reflective writing. Next, the RJ scale’s item formulation was based on the newly developed scheme. The instrument was distributed to a pilot group (38 students) to check for the overall clarity of the items; however, no changes were made because the pilot group participants reported no problems regarding the clarity of the scale’s items. The sample included 19 Education students and 19 Health students (80% females). To this end, convenience sampling was used–a non-probability sampling. The participants have voluntarily chosen to participate as a part of the pilot group. This type of sampling is most useful for pilot testing (Creswell and Plano Clark, 2011; Zhou, 2019).

To formulate items related to transfer of learning, two statements were phrased. Next, to ascertain the structural validity and reliability of the newly developed questionnaire data were collected from pre-service teachers and Health Management students.

The pre-service teachers were enrolled in a mandatory two-semester course. The course included lectures and two group assignments. In the current course, 75 students were enrolled in the class. Regarding the pre-service teachers’ tasks, during the first month of the first semester, the RJ was mandatory but unstructured. In the second month, the instructor initiated a discussion about reflective writing and emphasized that the journals could help students perform better, learn more effectively, organize their thoughts and emotions, and track and evaluate their progress throughout the course. Students were told that reflective learning is about contemplating on what, why, and how they are learning (Waggoner-Denton, 2018). A semi-structured RJ was provided, including several prompt questions, which the students could choose from to answer in each entry (Learning to Learn Project, 2002; Assessment Tools for Higher Education Learning Environments [ASSET], 2020). These questions aimed to lead students to pinpoint the problems they were encountering during the learning process and to consider plans and remedies to solve them (e.g., “what strategy did you apply in learning this topic?”; “how can you make this strategy more effective?”; or “what techniques can you use to link your learning to prior knowledge and skills?”). Detecting personal blind spots and mental mind traps were also considered when developing the prompts (e.g., “describe the problems and challenges you had as a learner during the course or assignment”; or “evaluate the things about yourself you would like to improve”).

Yet, students could ponder upon things that had the greatest personal significance to them and freely convey them in writing. During the course, the students submitted a journal entry after each lesson in the first semester (805 entries in total). Entries were typically a single paragraph in length. In the second semester, students were required to submit four journal entries throughout the course. In this phase, an additional set of prompt questions was used, inviting students to think of learning experiences related to their future professional career or personal lives, using the above-mentioned semi-structured RJ method. During the second semester, a total of 264 entries were submitted. The instructor reviewed the entries after each lesson and ensured that each student had received at least two sets of feedback during the semester. Entries were typically three to four short paragraphs in length.

Concerning the health management course, 78 students of a Management of Health Service Organizations program (covering patient-doctor relations, quality of service in the healthcare system, and ethics and patient rights) were enrolled in a third-year course entitled “Assimilation of service quality in health systems” during the first semester. The course included lectures and two group assignments. The students were presented with a problem relevant to their course content, dealing with *accreditation*. The students in this research were asked to argue for or against the implementation of the accreditation process within hospitals. In the first assignment, the participants were asked to detail five arguments to establish their decision by using a concept map. In the second assignment, relying on the materials taught in their courses, the students were asked to obtain the necessary supporting information to substantiate their arguments. The students were asked to relate to their personal learning process by writing a RJ in which they were instructed by the lecturer to write about their self-perceived progress from their preliminary argument to a more complex one and describe their challenges and gains in light of the experience. Students were required to write and submit four journal entries throughout the course by using a set of prompt questions using the above-mentioned semi-structured RJ method (243 entries in total were obtained). The instructors in both courses were trained by Erasmus+ project’s experts (Assessment Tools for Higher Education Learning Environments [ASSET], 2020). This project was aimed at adapting learning and assessment methods to different courses in higher education settings. Therefore, the content and activities in each course were not identical, as each lecturer could employ an activity according to his/her course objectives and use RJs to encourage reflective writing among students.

### Data Analysis

In this study we used a mixed-method exploratory sequential research design. The qualitative data analysis was used to analyze the gathered materials and identify meaningful categories (Glaser and Strauss, 1967). Drawing on the deductive approach, a categorical scheme suggested by the theoretical framework was used (Learning to Learn Project, 2002). The inductive approach enabled identifying additional meaningful categories.

The journal entries (1312 in total) were reviewed, and their content was analyzed by four experts in the research field of health management, constructive learning, and assessment for lifelong learning. Inter-rater Cohen’s Kappa (k) reliability (Cohen, 1960) was used. Based on this analysis, a theoretical scheme was designed. This step led to the formulation, addition, subtraction, and adaptation of items related to the identified categories. All item descriptions without consensus were excluded from the analysis. Descriptions that were identified as unclear or too similar to another description were omitted. As a result, the number of descriptions was reduced from 46 to 31. Two single-item variables were formulated to measure the students’ tendency to report the use of RJs in their future personal and professional lives: “I will use reflective writing in my personal life,” “I will use reflective writing in my work (e.g., in my current or future classroom).” A single-item construct is permitted in Partial Least Squares-Structural Equation Modeling (PLS-SEM) (Hair et al., 2017) used in this research for data analysis. Following Diamantopoulos et al.’s (2012) guidelines, a single-item construct should be considered to be used (rather than a multi-item scale) in research with a small sample size.

To assess factor structure validity and internal consistency of the developed questionnaire, exploratory factor analysis and confirmatory factor analysis using PLS-SEM (Hair et al., 2017) with SmartPLS software were used. The latter technique was also used to assess the research hypotheses. This technique was chosen based on previous work (Hair et al., 2017) showing that PLS-SEM is a powerful method to analyzing models using small sample sizes and which overcomes problematic model identifications when small samples are used.

### Measurement Design and Evaluation

The journal entries (1312 in total) were reviewed, and their content was analyzed by four experts. Inter-rater Cohen’s Kappa (k) reliability (Cohen, 1960), which is commonly assessed in psychological research, was used. The raters were asked to check the theoretical categorization, and to identify descriptions relating to those categories, or identify new categories that emerge from the data. In each step, the *k* values were interpreted as follows, *k* < 0.20 poor agreement; 0.21 < *k* < 0.40 fair agreement; 0.41 < *k* < 0.60 moderate agreement; 0.61 < *k* < 0.80 good agreement; 0.81 < *k* < 1.00 very good agreement. Results of 0.61 < *k* < 1 were considered acceptable for the purposes of the current study. All descriptions without consensus were discarded from the analysis.

The content analysis of the RJ entries revealed a reflection scheme comprising two dimensions: the first refers to students’ current experiences, or “short-term related reflections.” This dimension deals with students’ in-process experiences during the course. The reflection included the following levels:

(1) Cognitive–relates to the content of the course, learning skills, and learning purposes.
(2) Behavioral–refers to the student’s behavior during the learning process.
(3) Affective (emotional)–pertains to emotions that arose during the learning experience.

The second dimension concerns long-term related reflections and includes students’ learning experience in relation to their future from the aspects of:

(1) Academic development.
(2) Professional development.
(3) Personal development.
(4) Multicultural development.

In addition, three essential metacognitive abilities were foregrounded within the scheme:

(1) Awareness of one’s learning experience.
(2) Evaluation of the learning experience.
(3) Regulation in attitude and behavior to perform better in the future.

Table 1 illustrates the scheme and presents excerpts from the students’ RJ entries that substantiate its dimensions.

**TABLE 1:** Reflective writing scheme and students’ exemplary reflections.

Dimension 1	Short-term related reflections		
	Awareness	Evaluation	Regulation
**Cognitive level**	We were asked to read a text by Victor Frankel and to note two main topics. The topics we chose were “The Existential Vacuum” and “The Search for Meaning.”	I didn’t assimilate what we should “throw out” and what we should keep. In other words, how to distinguish between what was insignificant and what was important. This is important in writing a study–knowing how to sort the material, to distinguish what is most or least important.	It was difficult for me to understand the material, but after I asked the lecturer during the assignment, I understood what I had to do and how to choose two main topics from the text. Maybe I should consult with the lecturer more often.
**Behavioral level**	At first it was difficult to divide the work. We decided to write down what needed to be done and then each person would say what she wanted to do and what she was good at.	The way we learned in the group was efficient. We knew what to do. We organized ourselves quickly. We met twice before presenting the assignment in class. We encountered a few problems even from the standpoint of language.	We learned the hard way that next time we have to plan the assignment down to the smallest detail and give everyone tasks to do.
**Emotions (Affective level)**	I felt that the group was taking advantage of me during group work on the project because I’m a perfectionist and tend to take on jobs myself.	I felt that way because I didn’t stand up for what I believed. It’s important to do that in a group. I wanted the project to succeed and I didn’t trust the other students to do their share.	I learned that I shouldn’t feel embarrassed or feel inferior. I have to ask when I don’t understand. It doesn’t have to be unpleasant when friends answer and explain things. When I feel better about asking questions, I’ll be able to contribute more to members of the group.

**Dimension 2**	**Long-term related reflections**		
	**Awareness**	**Evaluation**	**Regulation**

**Academic Development**	It’s harder for me to read about a topic in an article. I feel that little by little I’m learning to mark the important sentences in the article.	During the exercise in philosophy, the lecturer asked for rationale for our argument. This was easy for me because it connected to the course in “Educational systems” in which we did an exercise in which we had to present rationale for our opinion, and then present rationale for the opposing position.	As time went by, I understood the need to think about the processes in the lesson. I developed critical thinking while writing reflections. It helped me a lot in understanding the material. I discovered not only what I understood more or understood less, but also what helps me understand things well and what I need to do.
**Professional Development**	I learned how much reflection improves my learning. I know that when I become a teacher, I will teach reflection to my students. I will teach them how to write a reflection and see to it that they write a reflective diary.	I’m sure that I will take the skills I have learned such as critical thinking with me when I become a teacher, but I will adapt them to the young age of the children I want to teach.	I think that I can develop my writing and thinking abilities through learning. This is very important to my future as a teacher. There are lessons in which I feel I have lost track, but when I begin writing at the end of the lesson and draw a connection to examples in real life, it helps me keep on track and even understand things better.
**Personal Development**	I learned that it’s good for me to think about what I learned, and that I’m capable as well. It compelled me to think about what I understood and what I didn’t, and about why. I saw that it’s really good for me to devote 10 min to what occurred in class and to answer the questions because I feel that I am teaching myself. The amount of difficulties I have declined dramatically from the first to the second semester.	I went to speak with the lecturer after we finished showing the presentation in front of the class. I told her that I understood that I can do it, that I have something to say to the other members of m group, that my statements have power, and that people listen to me and that I felt strong.	I am thinking and beginning to use this technique as the father of my teenage children. Instead of getting angry I ask them to stop and think for a minute and write an explanation. I haven’t tried it yet, but it’s an idea.
**Multicultural Development**	This lesson was interesting for me, particularly because we talked about Christianity, how it was born. I am a Christian, so I was drawn to this lesson.	The group consisted of people from different sectors and therefore gave depth to the work. It gave me added value of learning about different cultures from each other. I learned that language and respect are viewed differently in Arab and Jewish culture.	Through difficulties we discovered that common explanations contribute to understanding the material and depth of learning among all members of the group (Jews and Arabs). Students who understand more, explain again and again in their own words, they try to look for other words (in their mother tongue) to explain, everyone listens to everyone else, and conducts a discussion. This undoubtedly contributes to understanding and clarifying the material. Finally, we became connected and established a positive and supportive study atmosphere. Because of this, we didn’t notice that we had been sitting together for a long time.

Based on this analysis, the Reflective Journal Scale (RJs) was constructed including 31 items along two sub-scales: short-term (16 items) and long-term (15 items). All items were scored on a Likert-type score ranging from 1 = *not true at all* to 6 = *completely true*. Tables 2, 3 show the scales of the RJs along with the items. The items of the scale were originally generated in Hebrew and subsequently translated to Arabic; yet, for the purpose of this manuscript, they were translated into English, and back-translated by professional translators.

**TABLE 2:** The short-term scale: factors, item descriptions, item loadings, and reliability results (Cronbach’s alpha) *N* = 141.

No.	Writing in the RJ enabled me to…	Cognitive level	Behavioral level	Affective level
A5	Evaluate what additional material I need to learn in order to succeed in the course or assignment	**0.757**		
A6	Evaluate the purposes of the course or assignment (e.g., why do I need to learn the materials? what other purposes can be suggested)	**0.722**		
A7	Think of ways that can help me understand the material or assignment better	**0.709**		
A3	Describe why it is important to study the material or to complete the educational assignment (specify learning goals)	**0.694**		
A4	Evaluate my understanding of the assignment or materials taught	**0.691**		
A1	Describe exactly what I was asked to do in the course (or educational assignment)	**0.636**	0.423	
A2	Describe the main issues raised during the course (or educational assignment)	**0.632**		
A8	Think about the experiences I had during the course or assignment that improved my learning methods	**0.577**		
A10	Evaluate what worked well during the learning activity (e.g., during the group work)		**0.763**	
A9	Describe how I learned (the methods/activities I used to learn the subject)		**0.665**	
A12	To think whether the way I learned (for example, alone or in a group) is the best way to learn		**0.648**	
A13	Think about how to improve my ways of learning		**0.599**	
A11	Evaluate the problems that emerged during the learning activity (e.g., during the group work)		**0.593**	
A14	Describe my feelings during the course or assignment (What did I like most/least)			**0.852**
A15	Evaluate the reason for the feelings that emerged during the course or assignment (why did I feel that way?)			**0.821**
A16	Think of ways to improve my feelings about the course or assignment (e.g., about the learning material, the ways of learning)			**0.729**
*Cronbach’s alpha*		0.91	0.89	0.90

*Bold values indicate the highest loading for items corresponding to their respective factor.*

**TABLE 3:** The long-term scale: factors, item descriptions, item loadings, and reliability results (Cronbach’s alpha) *N* = 141.

No.	Writing in the RJ enabled me to…	Personal development	Professional development	Multicultural development	Academic development
A26	Evaluate what I learned from the experience during the course or assignment about my potentials (how far can I reach?)	**0.836**			
A25	Describe what I have learned from the course or assignment on the personal level–about myself (e.g., what are my strengths, what should be improved)	**0.724**			
A28	To think of ways I can use the insights about myself that have emerged in the course or assignment to become a better person in the future	**0.690**			
A27	Evaluate the things about myself I would like to improve	**0.628**			
A22	Describe how what I learned during the course (knowledge, skill) might help me (be useful) in my current or future work		**0.847**		
A23	Evaluate how it might be possible to adapt what I have learned during the course (knowledge, skill) to my current or future work (what should I add? what can I omit?)		**0.710**		
A24	Think about the experiences that I have “earned” during the course that might be useful in my current or future work		**0.677**		
A18	Describe the things that I found easy to carry out in the course or assignment–as a learner		0.562		**0.417**
A17	Describe the problems and challenges I had as a learner during the course or assignment		0.482		
A30	Evaluate things I have learned from a cultural perspective			**0.833**	
A31	Think about ways of linking the educational activity to the culture (language, customs, history) of students			**0.819**	
A29	Describe the relationship between different cultures and the studied materials			**0.799**	
A19	To describe what material or issues in the course or assignment might be related to my prior knowledge (for example, things I have learned in past courses)				**0.743**
A20	Evaluate what else can be done to better connect the learning material to my prior knowledge				**0.731**
A21	Think of ways that can help me deal with difficulties (remove the barriers) during learning activities				**0.545**
*Cronbach’s alpha*		0.89	0.89	0.91	0.86

*Bold values indicate the highest loading for items corresponding to their respective factor.*

Exploratory factor analysis is often used when the researcher has no expectations of the number or nature of the variables (Henson and Roberts, 2006). Accordingly, all items (*N* = 141) separately for each sub-scale were subjected to a principal axis factoring followed by a varimax rotation with an eigenvalue >1.00 as a criterion for determining the number of factors. The analysis of the short-term reflections sub-scale resulted in three factors, which accounted together for 63.20% of the variance. The results for the long-term reflections sub-scale showed four factors, which accounted together for 70.53% of the variance. Tables 2, 3 present the item loadings (>0.40) on each of the factors and the computed internal consistencies (Cronbach’s alpha) for each factor, indicating sufficient reliability within the factors. Based on this analysis, item 17 was omitted due to a low loading result on its respective factor.

**FIGURE 1:**
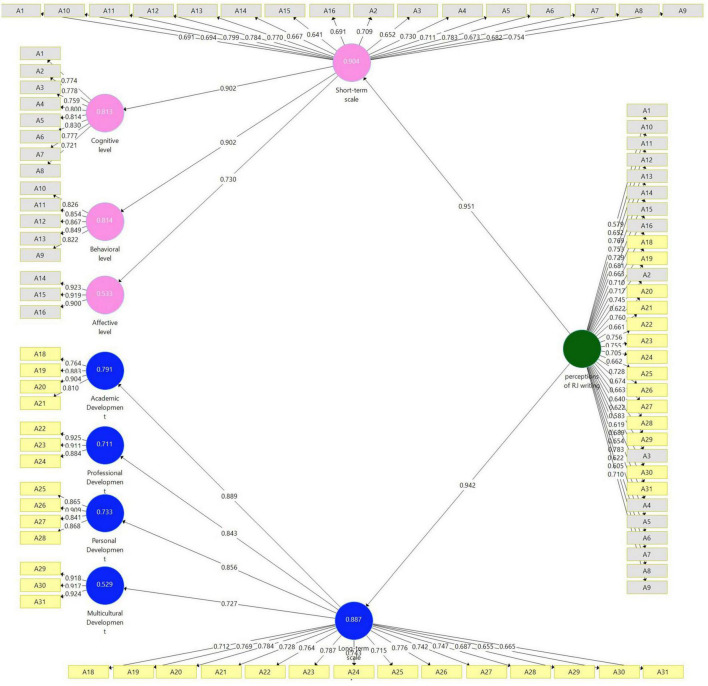
Model 1. Analysis results of the examination of the reflective measurement model by SmartPLS.

Partial Least Squares-Structural Equation Modeling was used to establish confirmatory validity for the RJs (*N* = 141). Given the complexity of the constructs, in this analysis a hierarchical component model (HCM, Hair et al., 2017) was designed (Model 1, Figure 1). Informed by the above-described exploratory factor analysis, this measurement model included the following lower-order components (LOCs): Cognitive, Behavioral, and Affective levels–which captured the subdimensions of the short-term related reflections (short term scale) higher-order component (HOC); and academic, professional, personal, and multicultural–which captured the subdimensions of the long-term related reflections (long term scale) HOC. The short- and long-term HOCs informed the perception of RJ writing factor. This model can be considered a reflective-reflective HCM type which indicates reflective relationships between the LOCs and the HOCs, and all first-order constructs are measured by reflective indicators. To represent the perception of RJ writing factor’s HOCs, all the indicators from the LOCs were assigned to them in the form of a repeated indicators approach (Hair et al., 2017, p. 283). Bootstrapping routine indicated significant positive links between all LOCs and HOCs (*p* = 0.000) ranging from β = 0.727 to β = 0.951.

#### Model Evaluation

The reflective measurement model assessment included composite reliability to evaluate internal consistency, individual indicator reliability (provided in Tables 2, 3), and average variance extracted (AVE) to evaluate convergent validity. The evaluation also included discriminant validity by using the heterotrait-monotrait (HTMT) ratio of correlation (Hair et al., 2017). Composite reliability takes into account the different outer loadings of the indicator variables, and varies between 0 and 1, with higher values indicating higher levels of reliability. Values of 0.60–0.70 are acceptable in exploratory research (Hair et al., 2017, p. 112). In the current analysis, the values ranged from 0.91 to 0.96.

Average variance extracted is defined as “the grand mean value of the squared loadings of the indicators associated with the construct” (Hair et al., 2017, p. 114). Thus, it is equivalent to the communality of the construct. An AVE value should be higher than 0.50 (i.e., the construct explains more than half of the variance of its indicators). AVE was calculated for the seven latent constructs (on the left, Figure 1) accompanied by their indicators and ranged from 0.612 to 0.846. Finally, HTMT was used to calculate the ratio of the between-trait correlations to the within-trait correlations. It is the mean of all correlations of indicators across constructs measuring different constructs. The threshold level of the HTMT should be below 0.90. HTMT was calculated for the seven latent constructs (on the left, Figure 1) and as shown in Table 4, all values were below 0.90.

**TABLE 4:** Results of discriminant validity by using the heterotrait-monotrait (HTMT) ratio of correlation (*N* = 141).

Factor	Academic development	Affective level	Behavioral level	Cognitive level	Multicultural development	Personal development
Affective level	0.712					
Behavioral level	0.807	0.690				
Cognitive level	0.659	0.510	0.758			
Multicultural Development	0.598	0.657	0.493	0.472		
Personal Development	0.787	0.578	0.613	0.619	0.527	
Professional Development	0.788	0.625	0.725	0.661	0.574	0.687
						

## Findings

**FIGURE 2:**
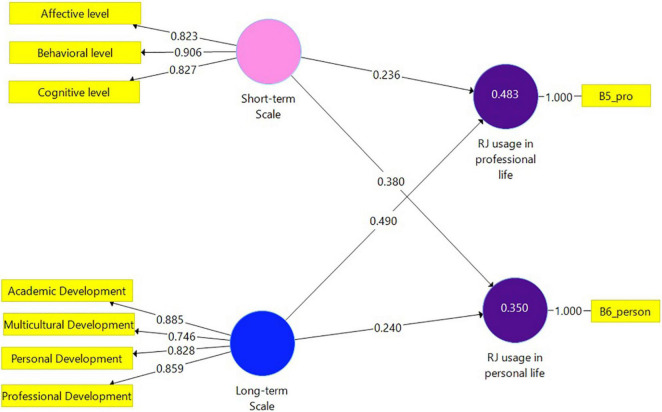
Model 2. Analysis results of the examination of the research hypotheses by SmartPLS (*N* = 141).

To assess *H1* and *H2*, Model 2 (Figure 2) was constructed for the total sample (*N* = 141). This parsimonious path model includes two independent constructs, represented in the model as cycles: The Short-term scale accompanied by its three indicators: Cognitive level, Behavioral level, and Affective level; and the Long-term scale with its four indicators: Personal Development, Academic Development, Professional Development, and Multicultural Development. The dependent constructs are RJ usage in professional life, and RJ usage in personal life. Relationships between the constructs as well as between the constructs and their assigned indicators are shown as arrows. In PLS-SEM, single-headed arrows, as shown between the constructs, are considered predictive relationships, and with strong theoretical support, can be construed as causal relationships. As illustrated in Figure 2, paths were specified based on the proposed assumptions.

Table 5 presents the bootstrapping routine results of the direct effects (Model 2). Both dependent variables (RJ usage in professional life and RJ usage in personal life) were positively explained by the independent variables. The highest coefficient result was shown between the Long-term scale and RJ usage in professional life, the lowest was detected between the Short-term Scale and RJ usage in professional life. *H1* and *H2* were confirmed.

**TABLE 5:** Significance analysis of the direct effects for each model.

Path	Direct effect	*T* statistics	*p* value
**Model 2**			
Long-term Scale → RJ usage in personal life	0.240	2.114	0.035
Long-term Scale → RJ usage in professional life	0.490	5.045	0.000
Short-term Scale → RJ usage in personal life	0.380	3.500	0.001
Short-term Scale → RJ usage in professional life	0.236	2.029	0.043
**Model 3**			
Long-term Scale → RJ usage in personal life	0.263	2.560	0.011
Long-term Scale → RJ usage in professional life	0.439	5.407	0.000
Short-term Scale → RJ usage in personal life	0.438	4.084	0.000
Short-term Scale → RJ usage in professional life	0.323	3.244	0.001
**Model 4**			
Long-term Scale → RJ usage in personal life	0.518	2.771	0.006
Long-term Scale → RJ usage in professional life	0.694	3.618	0.000
Short-term Scale → RJ usage in personal life	0.118	0.662	0.508
Short-term Scale → RJ usage in professional life	0.095	0.458	0.647

### Model 2 Evaluation

Collinearity was examined by the variance inflation factor (VIF) values of all sets of predictor constructs in the structural model. The results showed that the VIF values of all combinations of endogenous and exogenous constructs are below the threshold of 5 (Hair et al., 2017) ranging from 1.00 to 2.52. The coefficient of determination (*R*^2^) value was also examined. *R*^2^ for RJ usage in professional life (0.483) and RJ usage in personal life (0.350) can be considered moderate (Hair et al., 2017). In addition to measuring the *R*^2^ values, the change in the *R*^2^ value when a specified exogenous construct is omitted from the model was used to evaluate its impact on the endogenous constructs. This measure is referred to as the *f*^2^ effect size when values of 0.02, 0.15, and 0.35, respectively, represent small, medium, and large effects. Small effect size results were indicated between the Long-term Scale and RJ usage in personal life (0.031), the Short-term Scale and RJ usage in professional life (0.038), and between the Short-term Scale and RJ usage in personal life (0.078). A large effect was indicated between the Long-term Scale and RJ usage in professional life (0.162). Finally, the blindfolding procedure was used to assess the predictive relevance (*Q*^2^) of the path model. Values larger than 0 suggest that the model has predictive relevance for a certain endogenous construct (Hair et al., 2017). The *Q*^2^ value for RJ usage in professional life was 0.470 and for RJ usage in personal life *Q*^2^ = 0.332.

**FIGURE 3:**
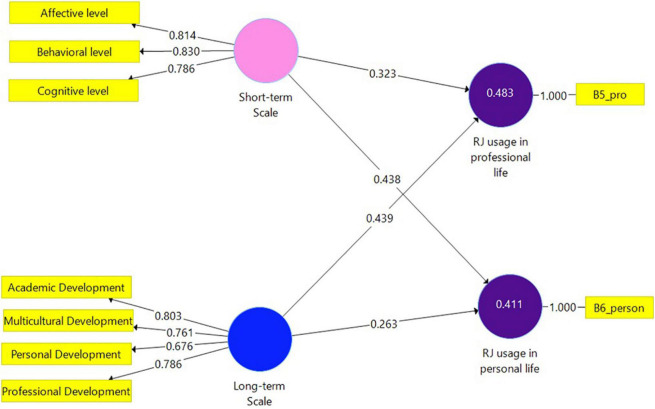
Model 3. Analysis results of the examination of the research hypotheses by SmartPLS (pre-service teachers sample *N* = 75).

To assess *H1* and *H2* for the pre-service teachers’ data Model 3 (Figure 3) was constructed. This model is identical to Model 2, however, includes data gathered from pre-service teachers. Table 5 illustrates the results of this analysis. Both dependent variables (RJ usage in professional life and RJ usage in personal life) were positively and significantly explained by the independent variables. The highest coefficient result was shown between the Long-term scale and RJ usage in professional life.

### Model 3 Evaluation

Variance inflation factor values of all combinations of endogenous and exogenous constructs were found below the threshold of 5 and equal to 1.751. *R*^2^ for RJ usage in personal life (0.411) and *R*^2^ for RJ usage in professional life (0.483) can be considered moderate. Small effect size results were indicated between the Long-term Scale and RJ usage in personal life (0.067), and between the Short-term Scale and RJ usage in professional life (0.116). Large effects were indicated between the Short-term Scale and RJ usage in personal life (0.186), and between the Long-term Scale and RJ usage in professional life (0.212). The *Q*^2^ value for RJ usage in professional life was 0.436 and for RJ usage in personal life *Q*^2^ = 0.360.

**FIGURE 4:**
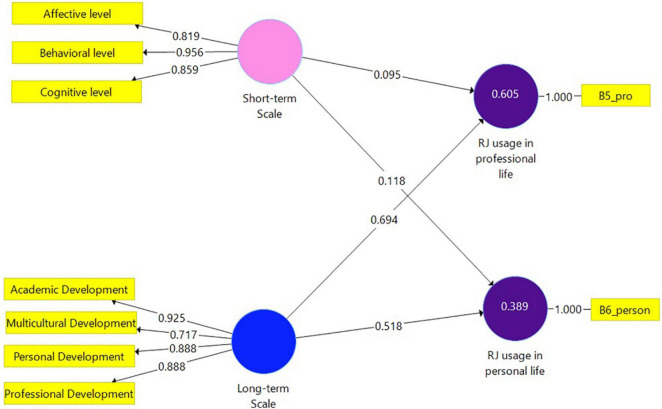
Model 4. Analysis results of the examination of the research hypotheses by SmartPLS (Health Management sample *N* = 66).

To assess *H1* and *H2* for Health Management students Model 4 (Figure 4) was constructed. This model is identical to Model 2, however, includes data gathered from Health Management students. Table 5 illustrates the results of this analysis. The dependent variables were significantly informed by the Long-term scale. Other coefficient results were found non-significant.

### Model 4 Evaluation

Variance inflation factor values of all combinations of endogenous and exogenous constructs were found below the threshold of 5 and equal to 4.040. *R*^2^ for RJ usage in personal life (0.389) and *R*^2^ for RJ usage in professional life (0.605) can be considered moderate. Small *f*^2^ effect size results were indicated between the Short-term Scale and each dependent variable and were equal to 0.006. A higher result was obtained between the Long-term scale and RJ usage in personal life (0.109). A large effect was indicated between the Long-term scale and RJ usage in professional life (0.302). The *Q*^2^ value for RJ usage in professional life was 0.567 and for RJ usage in personal life *Q*^2^ = 0.338. *H1* and *H2* were corroborated.

## Discussion

The present study’s overarching goal was to develop and validate a measurement to assess higher education students’ perceptions of RJ writing and their intention to transfer the knowledge and skills they acquired through the process to their work environment and/or personal lives. It also aimed to provide scores supporting evidence of reliability and validity that captures theoretical and new components of the construct. Drawing on the deductive approach, the qualitative data analysis was found to corroborate the dimensions suggested by the theory, i.e., the two timelines of reflections regarding the course assignments (the short-term scale) and those related to the students’ expected future development (the long-term scale).

Based on the inductive approach, pertaining to the short-term dimension, the current study elaborates on previous research by suggesting incorporating reflective writing in the learning process that provides students with opportunities to reflect on emotional aspects they experience during their learning. Previous and contemporary psychological studies on emotions in the fields of education and health professions (Mortari, 2015; Huang et al., 2020; Barbagallo, 2021; Karnieli-Miller et al., 2021; Szenes and Tilakaratna, 2021) underscored the educative significance of affective self-assessment which is a reflective practice that enables students to explore and gain awareness of their own emotions. This practice includes managing emotions with self-regulating strategies. The present study adds to the corpus of knowledge by suggesting the use of RJ writing as a tool that enables students to reflect on emotions during their learning process. As illustrated by this study’s empirical model, emotional reflections are considered part of students’ in-process experiences during the course and may increase their tendency to use such reflective practices during their personal lives. Mortari (2015) explained this connection by stating that “educating a person to care for himself/herself is educating that person to understand the emotional life of the mind” (p. 159). Therefore, as suggested by this research, RJs can be considered a vital pedagogical tool that enables students to reflect on their emotions during their studies and to further integrate this practice into the fabric of their everyday lives.

Regarding the long-term scale, beyond the three contextual aspects found in theory (academic, professional, and personal), the analysis also revealed a contextual element referring to the development of a student’s multicultural openness. This element of accepting the “other” is considered an integral part of any discussion of lifelong learning (Alt and Raichel, 2018) and is typically treated as a desired learning outcome of higher education (Bowman, 2014). In the current research, multicultural students comprised the sample, therefore, it might be inferred that in such diverse learning environments, students may benefit from practices that enable them to reflect on cultural aspects related to their learning. According to the empirical model, this practice is perceived to be part of students’ long-term related reflections which include students’ learning experience in relation to their future and may increase their propensity to transfer reflective skills to their professional lives. This premise can be strengthened by Rushton and Duggan (2013) who suggested using reflective practices to enable multicultural students to engage more effectively in their personal and professional development. RJ writing is considered a “safe” practice where multicultural students felt they were able to openly communicate cultural challenges they encountered. Another pivotal aspect is the importance of incorporating reflection into the education of teachers, who may use this valuable practice in their future learning environments when working with diverse students and families (Miller-Dyce and Owusu-Ansah, 2016).

The quantitative analysis corroborated the validity of the theoretical scheme of both scales (the long-term and short-term scales alike), in two different samples of pre-service teachers and health managers. Hence, whereas previous theoretical studies suggested some of the reflective dimensions, this study comprehensibly corroborated a holistic scheme incorporating cognitive, behavioral, and affective short-term and long-term related reflections, including students’ learning experience with relation to their future academic, professional, personal, and multicultural development.

In line with previous research suggesting that reflective writing may lead to a greater transfer of knowledge and skills to future settings (Brown et al., 2011; Griggs et al., 2018), it was expected that students’ positive perceptions of the reflective writing skills they had gained during the process would be positively connected to their tendency to transfer the skills to their professional and personal lives. According to the PLS models, pre-service teachers and health managers mainly underscored the importance of long-term reflective skills for their tendency to use them in their future professional lives. This implies that encouraging students to think of a learning experience in the context of their long-term development might increase their tendency to use reflective skills in their future careers. Thus, it might be inferred that having students reflect on their learning experiences and, more importantly, discover how these experiences might be linked to their intrapersonal and interpersonal development (National Research Council, 2012) might help them recognize the benefit of this tool and encourage them to transfer RJ skills to future activities.

### Limitations and Recommendations for Future Research

Several limitations and directions for future research warrant mentioning. First, this study offers a new measurement scale that captures students’ perceptions of reflective writing skills. Future studies could further substantiate its validity by showing how it might be connected to scores from another instrument designed to assess a construct it would theoretically be related to; for example, deep approaches to learning (Biggs et al., 2001). Second, in this study, students’ perception of transfer, and not their actual behavior, was measured; thus, observations of activities in a practical workplace setting were not gleaned. Based on the theory of planned behavior (Ajzen, 1991) one may expect individuals to behave based on their pre-existing attitudes and behavioral intentions. However, future work should consider attaining observational data to further strengthen these assumptions. Third, the three dimensions of awareness, evaluation, and regulation, suggested by theory (Magiera and Zawojewski, 2011) and foregrounded in the qualitative analysis conducted in the current study, were not validated in the quantitative analysis. The use of unrestrictive approaches such as structural similarity analysis could help reveal insights often overlooked by classical factor analysis methods (Tucker-Drob and Salthouse, 2009), and are therefore recommended to uncover these dimensions.

Fourth, this study was conducted in a single country and was limited to health and education students; therefore, the results cannot necessarily be generalized to students of other regions and study tracks. Cross-cultural validation of the results is required to corroborate the structural and measurement models introduced in this study.

## Conclusion and Implications

With the paucity of empirical work, the overarching aim of this research was to design a generic reflection scheme for identifying dimensions of reflective thinking and to validate a questionnaire to measure students’ perceptions of their reflective writing experiences. Lastly, this study sought to assess the connection between perceived reflective writing and students’ tendency to transfer this skill to their future careers and personal lives. The newly developed questionnaire items might collectively offer a bank of prompting questions organized in a validated theoretical scheme. Teachers may choose statements from the newly designed scale and formulate them as questions to inspire and assess different levels of reflective thinking in their students in line with the learning outcomes set by them. The questions can assist teachers in structuring the journals and helping students correctly understand and carry out the process of reflective thinking.

This study may also mitigate the main barrier in using RJ practices as formative assessment tools. RJ has been identified as a time-consuming task for teachers who need to invest extra time in checking and feedbacking such assignments when large groups of students are involved. Feedbacking is essential for students as it helps them identify their strengths and weaknesses during the learning process, while also being beneficial for teachers, who can use this information to adapt their instructional strategies to different ability learners (Alt and Raichel, 2021). To tackle this challenge, teachers may use the proposed scheme developed in the current study and gradually administer the questions provided. For instance, during a continuous assignment, they can ask their students to submit several entries, each relating to a different level or dimension indicated in the scheme. Moreover, online learning environments could offer opportunities for reflection, helping students to focus on learning and to guide their engagement in reflection (Saito and Miwa, 2007; Cheng, 2017). Reflection could be also combined with peer review (a form of reflective learning based on the theory of experiential learning, Kolb, 1984) and peer feedback to positively affect students’ SRL outcomes (van den Boom et al., 2007; Yang, 2010).

Reflective writing has been identified as an effective pedagogical tool to spur students’ flexibility, adaptability, planning ability, and self-regulation of learning. These capabilities are becoming an essential and inseparable part of the array of tasks that characterize professionals in the 21st century. The current study’s suggested validated generic scheme can be adapted and integrated into different curricula, thereby possibly increasing the potential of infusing RJ instructional strategies into higher education curricula, improving the quality of reflection in student journals, and promoting lifelong learning skills.

## Data Availability Statement

The datasets presented in this study can be found in online repositories. The names of the repository/repositories and accession number(s) can be found below: doi: 10.17632/8sndpz4s8k.1.

## Ethics Statement

The studies involving human participants were reviewed and approved by Kinneret College Ethics Committee. The patients/participants provided their written informed consent to participate in this study.

## Author Contributions

DA: conceptualization, data curation, writing–original draft preparation, and writing–reviewing and editing. NR: conceptualization, data curation, methodology, writing–original draft preparation, and writing–reviewing and editing. LN-S: data curation, methodology, and writing–original draft preparation. All authors contributed to the article and approved the submitted version.

## Conflict of Interest

The authors declare that the research was conducted in the absence of any commercial or financial relationships that could be construed as a potential conflict of interest.

## Publisher’s Note

All claims expressed in this article are solely those of the authors and do not necessarily represent those of their affiliated organizations, or those of the publisher, the editors and the reviewers. Any product that may be evaluated in this article, or claim that may be made by its manufacturer, is not guaranteed or endorsed by the publisher.
